# Dietary and Endogenous Advanced Glycation End Product and Breast Carcinogenesis: A Challenging Lack of Association in a Rigorous Case–Control Study

**DOI:** 10.1155/jnme/6687630

**Published:** 2026-07-31

**Authors:** Sadaf Alipour, Ramesh Omranipour, Alireza Abdollahi, Behnaz Jahanbin, Bardia Gholami, Sakineh Shab-bidar, Amirnader Emami-Razavi, Maryam Selk-Ghaffari, Foroozan Ghalkhani, Azin Saberi, Zahra Morovati, Marzieh Orouji, Maryam Haghighi, Mahtab Vasigh, Elham Nazar, Samareh Heydari, Sareh Saien, Bita Eslami

**Affiliations:** ^1^ Breast Diseases Research Center, Cancer Institute, Tehran University of Medical Sciences, Tehran, Iran, tums.ac.ir; ^2^ Department of Surgery, Arash Women’s Hospital, School of Medicine, Tehran University of Medical Sciences, Tehran, Iran, tums.ac.ir; ^3^ Department of Surgical Oncology, Cancer Institute, School of Medicine, Tehran University of Medical Sciences, Tehran, Iran, tums.ac.ir; ^4^ Department of Pathology, Imam Khomeini Hospital Complex, Tehran University of Medical Sciences, Tehran, Iran, tums.ac.ir; ^5^ Department of Pathology, Cancer Institute, Imam Khomeini Hospital Complex, Tehran University of Medical Sciences, Tehran, Iran, tums.ac.ir; ^6^ Faculty of Medicine, Shahid Beheshti University of Medical Sciences, Tehran, Iran, sbmu.ac.ir; ^7^ Department of Community Nutrition, School of Nutritional Sciences and Dietetics, Tehran University of Medical Sciences, Tehran, Iran, tums.ac.ir; ^8^ Iran National Tumor Bank, Cancer Institute, Tehran University of Medical Sciences, Tehran, Iran, tums.ac.ir; ^9^ Sports Medicine Research Center, Neuroscience Institute, Tehran University of Medical Sciences, Tehran, Iran, tums.ac.ir; ^10^ Department of Nursing, Arash Women’s Hospital, Tehran University of Medical Sciences, Tehran, Iran, tums.ac.ir; ^11^ Department of Nursing, Imam Khomeini Hospital Complex, Tehran University of Medical Sciences, Tehran, Iran, tums.ac.ir; ^12^ Department of Surgical Oncology, Hospital-Fox Chase Cancer Center, Temple University, Philadelphia, Pennsylvania, USA, temple.edu; ^13^ Department of Pathology, Sina Hospital, Tehran University of Medical Sciences, Tehran, Iran, tums.ac.ir; ^14^ Faculty of Medicine, Tehran University of Medical Sciences, Tehran, Iran, tums.ac.ir

**Keywords:** advanced glycation end product, breast cancer, exercise, nutrition, premenopause

## Abstract

**Introduction:**

Advanced Glycation End (AGE) Product is produced by atypical metabolism when food is cooked at high temperatures in a short time, such as roasted foods, or when chemicals are added as in processed foods. Research on the association between AGE and breast cancer (BC) has been inconclusive.

**Methods:**

This multicenter case–control study investigated the relationship between serum AGE (sAGE), tissue AGE (tAGE), and dietary AGE (dAGE) and BC, considering physical activity (PA). The study included 44 hormone receptor–positive premenopausal BC patients and 42 controls. Data on diet and PA were collected using validated Persian questionnaires. sAGE and tAGE were measured using blood and tissue samples.

**Results:**

The dAGE was 16,104.98 ± 6542.04 and 15,084.53 ± 5827.69 kilo‐units/day, while PA was significantly lower in cases than controls (773.64 ± 829.92 vs. 1920.95 ± 2999.91 MET‐minutes/week, *p* = 0.02). The mean sAGE was 626.11 ± 695.00 ng/mL in cases and 713.93 ± 829.67 in controls. tAGE was positive in 34.1% and 28.6% of cases and controls.

**Conclusion:**

We found no association between dAGE and BC or dAGE and sAGE or tAGE. PA was less in the BC group but unrelated to sAGE and dAGE. As we accounted for all relevant variables, our negative findings provide strong evidence against an association between AGEs in diet, PA, and BC.

## 1. Introduction

Advanced glycation end product (AGE) in the human body is the product of glycation of proteins or lipids. In normal conditions, proteins undergo glycosylation. However, in hyperglycemic or oxidative stress conditions or during aging, glycation occurs, and irreversible abnormal glycated products are derived that interfere with normal cellular activities [1, 2].

Diet high in processed food or food that is cooked at high temperatures in a short time (as roasted food) is an exogenous source for AGE [3, 4]. Also, using a high‐calorie diet induces endogenous AGE production [2]. The amount of AGE in the body may also be affected by physical activity (PA), and increased PA might lower body AGE levels [5].

An elevated AGE is associated with various disorders including diabetes, heart diseases, and neurological conditions. Studies about the association of AGEs and cancers have been carried out in recent years. In the “European Prospective Investigation into Cancer and Nutrition (EPIC)” cohort, Cordova et al. [6] did not find any association between dietary AGE (dAGE) and the risk of cancer overall, but detected a weak positive association with some cancers, and an indirect association with others. Studies about the relation of serum AGE (sAGE) and cancers also have shown variable results, but a very recent systematic review and meta‐analysis [7] could not find any association between sAGE and cancer incidence overall.

Some clinical studies have targeted the association of breast cancer (BC) and sAGE or tissue AGE (tAGE) levels [8], or dAGE [9, 10]; and the results are not uniform.

If increased levels of AGE are related to BC, and if this can be modified by PA and diet, this surely needs to be focused on. Thus, we carried out this study to investigate the association of serum and tissue levels of AGE and premenopausal hormone‐positive BC, and their association with PA and diet.

## 2. Materials and Methods

This is a case–control multicenter study held from January 2021 to December 2022. The study was approved by the Ethics Committee of Tehran University of Medical Sciences (TUMS) (Ethics Approval Code IR.TUMS.DDRI.REC.1399.039). All participants signed an informed consent. The project was run according to the ethical principles of the Declaration of Helsinki.

### 2.1. Eligibility Criteria

Participants were selected among the premenopausal women who attended the Breast Clinic of three academic hospitals affiliated with TUMS and a private breast cosmetic surgery office for the controls. The Breast Cancer Group (BCG) (case) consisted of women with newly diagnosed BC, and the Healthy Breast Group (HBG) (control) involved women without any breast disease who underwent cosmetic breast surgery.

For the BCG, the other inclusion criteria involved a newly diagnosed, Tomor size > 10 mm, early, hormone receptor–positive, invasive carcinoma of no special type that underwent upfront breast surgery. For the HBG, the criteria were the absence of a self‐ or family history of BC and the absence of any breast disease in the preoperative assessment.

Exclusion criteria for all participants comprised the past or recent history of other cancers, diabetes, Alzheimer’s, cardiovascular or cerebrovascular disease, rheumatologic disease, and hepatic, renal, or cardiac failure. In addition, T4 and N3 tumors (according to the American Joint Committee on Cancer’s Staging System–AJCC TNM classification of BC) [11] in the BCG, women in the HBG who had a pathology in the surgical specimens, and all participants with incomplete questionnaires were excluded from the study.

### 2.2. Settings and Sampling

An experienced staff was trained in each of the study centers by the research nutritionist about how to correctly fill the food frequency questionnaires (FFQ), by the sports medicine specialist for filling the Global Physical Activity Questionnaire (GPAQ) and measuring body contour indices, by the chief pathologist and the director of the tumor bank about the method of gathering and transferring samples. A previously validated FFQ [12] and GPAQ [13–15] in the Persian language were used.

At the point of entry into the study, women were interviewed by the trained staff, and three forms were filled for every participant: the main form which consisted of personal, anthropometric, and demographic data, reproductive history, and self‐ or family medical history; the FFQ, for assessing the past year’s diet, and the GPAQ.

A 5 mL blood sample was collected from all participants before anesthesia in the operating room. The samples were centrifuged at 3000 rounds per minute (RPM) for 10 min. The sera were separated and immediately transported in ice bags to the National Tumor Bank and stored in nitrogen vapor at −190°C until further analysis.

A sample of tumoral tissue and a sample of healthy tissue far from the tumor were taken from the BCG, and a random sample of breast tissue was taken from the HBG.

### 2.3. Variables and Outcomes

The main variables of this study were sAGE in both groups, the tAGE in all tissue samples, the dAGE and PA in both groups. Additionally, participants’ age, body mass index (BMI), waist and hip circumference, and reproductive features were assessed. Fasting serum glucose levels, and renal and liver function tests were checked in all participants.

Our primary outcome was the difference in sAGE and tAGE between the two groups. Our secondary outcomes included changes in sAGE or tAGE according to dAGE and PA as well as the difference of tAGE in normal and tumoral breast tissue in the BCG.

## 3. Measurements

### 3.1. Tissue Microscopic Examination and Tumor Staging

The histologic type of the breast tumor was diagnosed on histological examination according to the World Health Organization (WHO) Classification of Tumors of the Breast, fifth edition (2019) [16]. Hormone receptor status was described by immunohistochemistry (IHC) assessment, and estrogen receptor positivity was defined as more than 1% staining. Tumor stage was considered based on the TNM Classification of Malignant Tumors of the Union for International Cancer Control (UICC) [17]; stage 1 and 2 tumors were entered in the study if they were planned to undergo upfront surgery by the treating medical team.

### 3.2. AGE Levels in Serum

Measurement of AGE levels in serum was conducted using kits from Crystal Day Co., Ltd, China. These kits use a double‐antibody sandwich enzyme‐linked immunosorbent assay method. The test was carried out according to the kit’s instructions, with an assay range of 0–4000 ng/L and a sensitivity of 5.23 ng/L.

### 3.3. AGE Levels in Tissue

First, a block of normal breast tissue of the HBG was chosen; then, appropriate blocks of tumoral and nontumoral tissue from normal and tumoral specimens of BC patients were selected (to assess AGE in both healthy and tumoral tissue in BCG). IHC assay of the formalin‐fixed, paraffin‐embedded slides was carried out manually. IHC staining was performed using the following antibodies: anti‐AGE (AB9890 EMD Millipore polyclonal antibody, 1/100 dilution) followed by ready‐to‐use anti‐goat synthetic polymer (Master Diagnostica).

Paraffin‐embedded tissue was sectioned (3 μm), deparaffinized, and further unmasked by heat treatment in a pressure cooker for 25 min at 100°C in citrate buffer (pH 6). Staining was evaluated by the pathologist, and a score for staining intensity (0 = *no staining*, 1 = *weak-staining*, 2 = *intermediate-staining*, 3 = *strong-staining*) and for percentage of positive tumor cells (0 = 0%–4%; 1 = 5%–9%, 3 = 10%–49%, 4 = 50%–79%, 5 = 80%–100%) was determined. The final score or H‐score was calculated by multiplying the intensity and percentage scores; this scoring method was derived from the study of Nass et al. [18].

### 3.4. Dietary Intake of AGE

We used a valid semiquantitative FFQ to record the habitual dietary intake of participants [12]. The serving size of each food item consumed by participants was collected by a trained interviewer on a daily, weekly, monthly, or yearly basis. To extract the energy and nutrient content of food items, we used Nutritionist IV software (First Databank, San Bruno, CA, USA), modified for Iranian foods [19]. Due to the lack of information about dAGE in the Iranian Food Composition Table, we extracted data from the published food carboxymethyl lysine (CML)–AGE database for 549 routinely consumed food items for the Northeastern American multiethnic urban population, which was assessed by a validated immunoassay method [20, 21]. To maintain methodological consistency, enable comparability with earlier studies, and ensure comprehensive coverage of food items, the older validated database continues to be the recommended and most widely used resource in nutritional AGE research. AGE value was calculated per day, according to kilo unit (kU) amounts in 100 g solid food or 100 mL liquid. The values for some Iranian‐specific food items were estimated from similar food items. Some kinds of confectioneries, as there was no similar food in the database, were considered as missing. We also considered the mean values of comparable fruits and vegetables for the AGE values of all fruits and vegetables that were not available [22]. The final values of AGEs were divided by total energy intake (TEI) and considered as dAGE/total energy intake (dAGE/TEI).

### 3.5. Assessment of PA

Data on PA were extracted from the GPAQ, a questionnaire developed under direct observation of the WHO in 2002 which is an acceptable global subjective method for the assessment of PA [23]. Total PA level was calculated by summing work domain, travel domain, and leisure time PA level expressed as metabolic equivalents (METs) minutes/week values. Duration of high‐intensity activity questions (expressed as hours) was multiplied by eight METs, and duration of moderate‐intensity activities and walking were multiplied by four METs (Organization WH: GPAQ analysis guide. In Geneva; https://www.who.int/ncds/surveillance/steps/resources/GPAQ_Analysis _Guide.pdf, 2012 [accessed December 30, 2019]). Simultaneously, sedentary behavior was evaluated via GPAQ according to daily hours of sitting or reclining.

### 3.6. Bias

To avoid a detection bias, one ELISA kit was used for the measurement of all the sAGE levels, and antibodies for IHC of tAGE were purchased from a single provider. Also, assessments of AGE levels were performed by one pathologist.

### 3.7. Quantitative Variables

We defined tAGE both as a continuous variable (tAGE‐cont), including an H‐score from 1 to 15, and as a categorical variable (tAGE‐cat), defined as positive for H‐score ≥ 6 and as negative for H‐score < 6.

Other variables, including sAGE, dAGE, BMI, age, and others, were considered as continuous variables, and questions, such as use of oral contraceptive pills (OCP), were considered as yes/no categories.

### 3.8. Sample Size

We estimated our study sample size according to the study by Tesařová et al. [24]. Considering this study, a minimum of 15 samples per group was necessary based on the mean sAGE levels in both cases and controls (327 ± 119 vs. 225 ± 70.3) with an 80% power and an alpha level of 0.05. To measure tAGE and dAGE, as well as examine the sAGE in our study, we decided to triple the sample size based on the available budget. Therefore, the inclusion of 45 participants in each study group was considered.

### 3.9. Statistical Methods

All the analysis was performed by SPSS 24 (IBM Corp. 2016. IBM SPSS Statistics for Windows, Version 24.0. Armonk, NY: IBM Corp). Data are presented as mean (± standard deviation) and number (and percentages) for continuous and categorical variables, respectively.

The normality of variables was tested by the Kolmogorov–Smirnov test. The comparison of continuous variables between two groups was conducted by Student’s t‐test or Mann–Whitney test based on the normality of variables. Furthermore, the correlation between variables was assessed by parametric (Pearson’s) or nonparametric test (Spearman’s rho), when appropriate.

Finally, multivariate logistic regression analysis was performed to estimate the association between variables and BC. Variables (age, BMI, breastfeeding duration, OCP, total physical activity [TPA], and sAGE) were selected in the multivariable model on the basis of the association with BC in univariate analyses (*p*‐value ≤ 0.1). Additionally, since dAGE is the primary exposure of interest, it was included in the multivariable model as well. Given the small scale and wide distribution of TPA, sAGE, and dAGE, the odds ratios (ORs) were reported per 100‐unit increase to provide a more interpretable effect size. *p*‐value less than 0.05 was considered statistically significant.

## 4. Results

### 4.1. Participants and Descriptive Data

Overall, 95 participants, including 49 women in the BC and 46 in the HB group, were entered in the study. Among these, the FFQ and GPAQ forms had not been filled completely in 5 and 2 participants in the BC and HB groups, respectively, and the tissue specimen was not appropriate for AGE evaluation by IHC in one BC and one HB patient; these were withdrawn from the study. Therefore, 86 participants, including 44 women in the BC and 42 in the HB group, were considered in the final analysis. All the BCs were stage I or II, hormone receptor–positive tumors. The fasting serum glucose levels and renal and liver function tests were normal in all participants. The mean age was 41.17 ± 7.43. Demographic, anthropometric, and reproductive data of participants in the two groups are demonstrated in Table 1; there were no smokers. Age, breastfeeding, and BMI were significantly different between the two groups.

**TABLE 1:** Demographic, anthropometric, and reproductive features of participants in the two groups.

Variable		Healthy Breast Group		Breast Cancer Group		Total		*p*‐value
		*N*	Mean ± SD	*N*	Mean ± SD	*N*	Mean ± SD	
Age (yrs)		42	39.67 ± 8.40	44	42.67 ± 6.05	86	41.17 ± 7.43	0.02

BMI (kg/m^2^)		41	28.02 ± 4.74	42	29.38 ± 6.35	86	28.76 ± 5.62	0.03

Breastfeeding time (*m*)		41	14.11 ± 10.25	42	37.45 ± 27.41	83	25.92 ± 23.76	< 0.01

Gravidity (*n*)		42	2.10 ± 1.95	44	2.55 ± 1.78	86	2.83 ± 1.87	0.27

Age at menarche (yrs)		36	16.19 ± 17.84	43	13.14 ± 2.49	79	14.53 ± 12.18	0.27

**Variable**	**Group**	***N***	**Percent**	***N***	**Percent**	***N***	**Percent**	**p** **-value**

OCP use	No	35	83.3	25	58.1	60	69.8	0.11
	Yes	7	16.7	18	41.9	25	29.1	

Infertility	No	39	92.9	41	95.3	80	93.0	0.63
	Yes	3	7.1	2	4.7	5	5.8	

Education	HS or less	5	12.2	16	39.0	21	24.4	0.03
	HS grad	19	46.3	12	29.3	31	36.0	
	Uni. grad	17	41.5	13	31.7	30	34.9	

Abbreviations: BMI = body mass index, Grad = graduate, HS = high school, N = number, OCP = oral contraceptive pills, SD = standard deviation, Uni = university.

### 4.2. Serum and tAGE, dAGE, PA, and BC

Total PA, sedentary life, sAGE, and tAGE‐cont did not have a normal distribution (*p* < 0.05), whereas dAGE and dAGE/TEI were normally distributed (*p*‐value > 0.05). Comparisons of sAGE, tAGE‐cont, tAGE‐cat, dAGE, dAGE/TEI, and total PA between the two groups are shown in Table 2. Although the mean sAGE was higher in healthy women (713.93 ± 829.67) than BC patients (626.11 ± 695), this was not statistically significant (*p* = 0.11). Furthermore, tAGE‐cont and tAGE‐cat were not statistically different between the two groups. Total activity was significantly higher in the HB than BCG (*p* = 0.02).

**TABLE 2:** AGE levels in serum, tissue, physical activity, and diet of participants in the two groups.

Variable		Healthy Breast Group		Breast Cancer Group		*p*‐value
		*N*	Mean ± SD	*N*	Mean ± SD	
Serum AGE (ng/mL)		42	713.93 ± 829.67	44	626.11 ± 695.00	0.11

Tissue AGE^∗^		42	3.19 ± 3.09	44	3.39 ± 3.67	0.80

dAGE (KU in 100 g solid food or 100 mL liquid)/day		42	15,033.10 ± 5894.84	44	16,177.09 ± 6450.58	0.64

dAGE/TEI (kU/kcal)		42	5.10 ± 1.74	44	5.32 ± 1.75	0.66

Total activity (MET‐minutes/week)		42	1920.95 ± 2999.91	44	773.64 ± 829.92	0.08

Sedentary life (hours/day)		42	4.06 ± 2.18	44	4.52 ± 2.44	0.43

**Variable**	**Group**	**N**	**Percent**	**N**	**Percent**	**p** **-value**

Tissue AGE^∗∗^	Negative	30	71.4	29	65.9	0.58
	Positive	12	28.6	15	34.1	

*Note:* *p*‐value refers to *t*‐test or Mann–Whitney test in continuous variables, when appropriate. The chi‐square test was used for categorical tissue AGE.

Abbreviations: AGE = advanced glycation end product, dAGE = dietary AGE, KU = kilo unit, MET = metabolic equivalent of task, N = Number, SD = standard deviation, TEI = total energy intake.

^∗^Tissue AGE as continuous level.

^∗∗^Tumoral tissue of the Breast Cancer Group as a categorical variables, negative: H‐score < 6, positive: H‐score ≥ 6.

**FIGURE 1:**
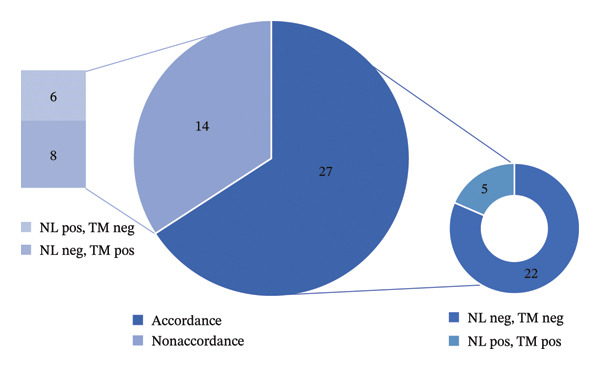
Comparison of tissue AGE (negative: H‐score < 6, positive: H‐score ≥ 6) in normal and tumoral tissue in the Breast Cancer Group. Pos = positive, Neg = negative, NL = normal, TM = tumoral.

We also evaluated whether tAGE was different in normal and tumoral tissue in the BCG; the results are shown in Figure 1. When considering tAGE‐cat, most cases were similar in their normal and tumoral tAGE. A negative tAGE in normal tissue with a positive tumoral tAGE occurred in only 8 (18.2%) of cases.

The result of logistic regression analysis showed that only 100‐MET minute of TPA had a borderline association (OR = 0.948, 95% CI: 0.901–1.007, *p* = 0.089) with BC; sAGE, tAGE, and dAGE/TEI did not show any association with BC in the final analysis (Table 3).

**TABLE 3:** Results of the logistic regression analysis.

Variables	Adjusted OR (95% CI)	*p*‐value
Age (yrs)	1.01 (0.93–1.10)	0.79
BMI (kg/m^2^)	1.02 (0.92–1.12)	0.77
BF (*m*)	1.06 (1.02–1.10)	0.003
OCP (yes/no)	2.16 (0.57–8.11)	0.26
sAGE	1.02 (0.96–1.09)	0.51
TPA	0.95 (0.90–1.01)	0.089
dAGE	1 (0.99–1.01)	0.71

Abbreviations: BF = breastfeeding duration in months, BMI = body mass index, CI = confidence interval, dAGE = dietary serum advanced glycation end products, OCP = oral contraceptive pills, OR = odds ratio, sAGE = serum advanced glycation end products, TPA = total physical activity.

## 5. Discussion

We performed a study to evaluate the association of premenopausal hormone‐positive BC and sAGE with consumption of AGE‐rich food, PA, and sedentary life. Considering the variability of results in the present literature, this study could clarify that AGE was not the main substance that linked diet and PA to BC.

This study had some strengths that provide a robust pivotal point among the controversies regarding AGE and BC. First, the tumors were all early hormone receptor–positive invasive carcinomas; our rationale was the positive association detected between these subtypes and dAGE [9] or tAGE [18] in previous studies. Second, patients were young, whereas higher levels of AGEs may be detected in older women [25–28]; age could have been the main confounding factor in previous studies. Third, all the participants were premenopausal, which implies a nearly similar hormone profile of participants and excludes the confounding effect of AGE levels related to ovarian dysfunction and menopause [10, 18, 29]. Fourth, we excluded patients with diabetes and other diseases associated with AGE, while these potentially confounding factors were not eliminated in some studies [18, 30, 31]; the normal fasting blood sugar in all the participants reinforces that hyperglycemia was not a driving factor for AGE formation.

### 5.1. BC, tAGE, and sAGE

Chiavarina et al. [8] retrospectively compared the amounts of a type of tAGE in 8 specimens of BC and normal breast tissue, and in cell lines. They detected higher levels in BC, but lower levels in triple‐negative BC. Walter et al. [5] retrospectively compared malignant with benign and normal breast tissue and sera from a tissue bank and found higher tAGE in hormone receptor–positive BC and higher sAGE in well‐differentiated and estrogen receptor–positive tumors. Nass et al. [18] retrospectively assessed tAGE in BC of various molecular subtypes. They found higher tAGE in age > 50 and in estrogen receptor–positive but not in progesterone receptor–positive BC and a lower tAGE in triple‐negative BC. Tesarova et al. [24] compared sAGE of 86 BC cases with 14 healthy women and found higher sAGE in the former group and in advanced BC cases. However, the existing studies are mainly retrospective studies carried out on preserved tissue from heterogenous cancer types, and the sample size of each subtype was not high. Interestingly, a recent prospective study on 32 BC patients and 32 controls by Alkan et al. [32] could not find a strong association between sAGE and inflammatory biomarkers in BC. Also, the recent meta‐analysis [7] excluded the relation between sAGE and cancer incidence. Overall, the consideration of main confounding factors in our study and the prospective nature as well as the precise uniform measurement of all variables and the results of more recent studies are in favor of our findings, which do not show any association between premenopausal hormone‐positive BC and tAGE.

### 5.2. BC and dAGE

In a recent prospective study, Jahromi et al. [33] studied the association of dAGE and BC in Iran. Although they revealed a positive association, the dAGE was not different among the cases and controls, and analysis of dAGE from different compounds also did not reveal any significant difference. The only significant finding was that women who were in the highest tertile of dAGE from oil sources were more prone to BC than the lowest tertile. It should be noted that there was a significant difference in other components of the dietary intake among the three tertiles, and the analysis was not adjusted for these differences. Also, various confounders including underlying diseases were not excluded. However, Omofuma [10] used the data of a large prospective cohort and did not find a significant difference between dAGE in postmenopausal BC patients and women with cardiovascular disease after correction for dietary details. Wada et al. [34] also evaluated the effect of dAGE on cancer through a large epidemiological prospective study, but did not find any association between dAGE and cancer overall, and BC specifically. The recent prospective study of Alkan et al. [32] also did not favor the association between dAGE and inflammatory markers in BC. Therefore, despite a few positive findings in the study of Kazemi Jahromi et al. [33], it seems that dAGE does not play a significant role in BC pathogenesis. This is in line with our findings. In addition, our study also considered the dAGE/TEI ratio and found no statistical difference between the groups.

### 5.3. BC, PA, and Sedentary Life

PA reduces the risk of BC [35, 36]; this effect may be limited to postmenopausal BC only [37, 38]. However, two studies [39, 40] reported a decline in sAGE secondary to a program including PA and healthy lifestyle. Also, Turner et al. [41] explain that the effect of PA on BC may be secondary to sAGE decrease. Walter et al. [5], after evaluating the link between BC and sAGE or tAGE, studied the effect of PA and a healthy diet on the sAGE of BC survivors; they showed a reduced sAGE secondary to the intervention. Nevertheless, our study shows that the effect of PA on BC rate reduction is not mediated through AGEs. To our knowledge, this is the first time that this matter has been studied by comparing healthy women and BC patients and considering the triple association of PA, AGE, and BC.

The diversity of the findings about the presence or type of association between AGEs and various cancers, including BC, has made challenging controversies. Also, some studies have focused on sAGE, some on tAGE, and some on dAGE. This is the first study that considered both serum levels and tissue levels of AGE in hormone‐positive premenopausal BC patients and in women with healthy breasts, compared normal and tumoral tissue in the cancer patients regarding AGE levels, and investigated the association of these issues with dAGE intake and PA. Therefore, our results are a valid proof for the lack of association between AGEs (including sAGE, tAGE, and dAGE) and BC in this specific group of patients although PA had an indirect association with BC, which was not mediated by AGEs [42].

## 6. Limitations

Our study had some limitations. First, AGEs are a broad class of compounds, and although the assays used in our study capture total AGEs, the possibility that a certain type of AGEs might be related to BC remains. Next, the temporal and causal relationship between these issues is unclear. Data collected via the FFQ and GPAQ are subject to recall bias and self‐measurement errors, e.g., patients might overestimate their activity in GPAQ; FFQs cannot capture cooking method details that affect AGE formation. Also, despite our considerations for an appropriate sample size, the relatively small sample may have limited the ability to detect minor associations. Consequently, theselimitations limitation may have contributed to the absence of a statistically significant relationship. In addition, while the homogeneity of the participants is a strength for the internal validity of our study, it limits the generalizability; so, the results may not apply to other demographics and dietary contexts [43].

## 7. Conclusion

In conclusion, our study evaluated the relationship of PA and dAGE with levels of AGE in hormone‐positive BC tissue of premenopausal women, normal tissue, and serum. Although PA reduced the rate of BC, AGEs were not related to this effect, because sAGE and tAGE levels did not correlate with PA levels in BC patients and in controls. Furthermore, dAGE did not affect sAGE and tAGE, and the levels of these variables were not different between the two groups. These findings suggest that AGEs do not play a major role in the diet‐PA‐BC relationship in this specific population. Nonetheless, considering the limitations of the study, larger prospective works are suggested to confirm and generalize this point.

## Author Contributions

All authors contributed to the study conception and design. Analysis of pathologic data was performed by Amirnader Emami‐Razavi, Behnaz Jahanbin, and Elham Nazar. Nutrition analysis was performed by Sakineh Shab‐bidar. PA analysis was conducted by Maryam Selk‐Ghaffari. Material preparation and data collection were performed by Bardia Gholami, Amirnader Emami‐Razavi, Foroozan Ghalkhani, Azin Saberi, Zahra Morovati, Marzieh Orouji, Maryam Haghighi, Mahtab Vasigh, Samareh Heydari, and Sareh Saien. Statistical analysis was conducted by Bita Eslami. The first draft of the manuscript was written by Sadaf Alipour and Bita Eslami, and all authors commented on previous versions of the manuscript.

## Funding

The research leading to these results received funding from the Industry Relations Office of the Research Deputy of Tehran University of Medical Sciences (TUMS) under grant agreement no: 99.3.210.50534.

## Disclosure

All authors read and approved the final manuscript.

## Ethics Statement

The study was approved by the Ethics Committee of Tehran University of Medical Sciences (TUMS) (Ethics Approval Code IR.TUMS.DDRI.REC.1399.039). All participants signed an informed consent. The project was run according to the ethical principles of the Declaration of Helsinki.

## Conflicts of Interest

The authors declare no conflicts of interest.

## Data Availability

The datasets used and/or analyzed during the current study are available from the corresponding author upon reasonable request.
